# Exploring Maternal Autonomy, Respect, and Satisfaction During Childbirth: Insights From Home Birth Experiences

**DOI:** 10.7759/cureus.91150

**Published:** 2025-08-27

**Authors:** Eleni Serpetini, Antigoni Sarantaki, Sofia Tzamaria, Maria Vlachou, Athina Diamanti

**Affiliations:** 1 Department of Midwifery, Faculty of Health and Care Sciences, University of West Attica, Athens, GRC; 2 Department of Midwifery, Elena Venizelou Hospital, Athens, GRC

**Keywords:** birth satisfaction, home birth, maternal autonomy, mistreatment in childbirth, respectful maternity care

## Abstract

Background

Maternal autonomy, respect, and satisfaction during childbirth are crucial aspects of maternity care. While hospital births are standard in many healthcare systems, home births are increasingly preferred by women seeking more personalized and autonomous experiences. This study evaluates maternal experiences of respect, autonomy, and satisfaction in home and hospital birth settings using validated assessment tools.

Methods

A retrospective study was conducted among 162 Greek women with at least one home birth experience. Data were collected via an online survey (2010-2023) using validated scales, including the Mother's Autonomy in Decision Making (MADM) scale, Mothers on Respect Index (MORi), Birth Satisfaction Scale-Revised (BSS-R), and Mistreatment in Childbirth Scale (MIST). Statistical analyses included the Kolmogorov-Smirnov test, the Mann-Whitney test, Spearman correlation, and multivariate regression to identify factors associated with satisfaction, autonomy, and experiences of mistreatment.

Results

Women reported significantly higher levels of respectful care during home births, as reflected in the MORi scores (mean: 74.4, SD = 8.8), compared to hospital births (mean: 56.4, SD = 13.7; p < 0.001). Measures of autonomy (MADM mean: 37.0, SD = 5.5) and birth satisfaction (BSS-R mean: 31.8, SD = 6.5) were also significantly higher among those who gave birth at home. Notably, the vast majority of participants (107, 88.4%) did not report any form of mistreatment, indicating generally positive childbirth experiences. Among the 14 (11.6%) who did report mistreatment, verbal abuse was most frequently mentioned (9, 7.4%), followed by privacy violations (2, 1.6%). Regression analysis highlighted two key factors that significantly contributed to greater birth satisfaction: the ability to choose one's birth position (p = 0.010) and receiving adequate information from midwives (p < 0.001).

Conclusions

Home births are associated with higher maternal autonomy, respect, and satisfaction compared to hospital settings. Maternity care policies should integrate respectful and autonomy-supportive practices to enhance maternal experiences in all birth environments.

## Introduction

The childbirth experience is one of the most transformative events in a woman’s life, leaving a lasting imprint on her physical, emotional, and psychological well-being. This multifaceted process extends beyond the physiological outcomes of delivery to encompass the care environment, the emotional and psychological support provided, and the degree of autonomy granted to women throughout the process [1,2].

Increasing evidence underscores the critical role of respectful and individualized care in shaping maternal satisfaction with childbirth. Maternal respect and autonomy are emerging as key determinants of positive birthing experiences, driving efforts to prioritize these aspects in maternity care policies and practices [3]. While hospital births remain the standard in most healthcare systems, there is a growing trend toward home births as an alternative that may better meet some women’s preferences for a more personalized and autonomous birthing experience [4].

Respectful maternity care, a concept rooted in the fundamental rights of women, ensures that all interactions during childbirth uphold dignity, allow informed decision-making, and provide a supportive environment for expressing concerns. The absence of such care has been linked to negative emotional outcomes, reduced trust in healthcare systems, and even traumatic childbirth experiences [5-7].

Tools like the Mistreatment in Childbirth Scale (MIST) have been developed to systematically assess experiences of disrespect and abuse during labor and delivery, offering valuable insights into areas where maternity care falls short in preserving women's dignity and autonomy [8]. In addition, tools like Mother’s Autonomy in Decision Making (MADM) [9] and Mothers on Respect Index (MORi) [10] have been developed to evaluate critical dimensions of care quality, including autonomy in decision-making and perceived respect during labor and delivery. These scales offer valuable insights into how care environments shape maternal experiences, providing actionable data for improving care practices. Similarly, the Birth Satisfaction Scale-Revised (BSS-R) assesses both the emotional and physical dimensions of satisfaction, offering a comprehensive measure of how women perceive their childbirth experience in relation to the care received [11].

Despite the growing body of literature on respectful maternity care, significant disparities persist between hospital and home birth settings in terms of respect, autonomy, and satisfaction. Hospitals, while equipped to manage medical complications, often operate within structured protocols that may inadvertently limit autonomy and personalized care. Conversely, home births often emphasize individualized care and shared decision-making, but they are not without their own challenges [12,13]. Understanding the nuanced differences between these two settings is crucial for identifying areas of improvement in maternal care across diverse contexts.

This study aims to bridge this gap by comprehensively analyzing maternal experiences of respect, autonomy, and satisfaction in both hospital and home birth settings. Using validated tools such as the MADM, MORi, MIST, and BSS-R scales, this research explores the interplay between care quality and maternal well-being. Furthermore, it investigates the impact of factors such as healthcare professionals’ communication, perceived mistreatment, and the freedom to make choices during childbirth on overall satisfaction and autonomy.

## Materials and methods

Study inclusion criteria

We conducted a retrospective study using the same cohort of Greek women with at least one home childbirth experience, initially recruited for validating the Greek version of the MADM scale [13] and the validation of Greek version of MORi [14]. Data collection was performed between January 2010 and December 2023 via an online survey.

Population studied

The inclusion criteria for the study cohort were: (1) women aged ≥18 years; (2) at least one home childbirth experience within Greece; and (3) agreement to participate in the study. The data set excluded incomplete survey responses and women who did not provide informed consent.

Data collection

We designed a self-administered, anonymized questionnaire using the Microsoft Forms (Microsoft Corp., Redmond, WA) electronic platform. The questionnaire's URL was shared on social media platforms, specifically within closed Facebook groups focused on women who have experienced home births and participated in parenthood discussions. To encourage participation from women who had given birth at home, a dedicated infographic was created and electronically distributed to relevant women's groups, organizations, and associations. Participation in the survey was entirely voluntary. Before beginning the questionnaire, participants were presented with a brief paragraph outlining the study's objectives and ensuring the confidentiality of their responses. Informed consent was obtained from all participants, and data collection was conducted anonymously.

The questionnaire included sections designed to comprehensively capture data on various aspects of maternal experiences. These include: a) Demographics: This section gathers information about participants' age, education level, family status, sexual orientation, religious beliefs, nationality, and other personal characteristics, enabling the analysis of how these factors influence childbirth experiences; b) Homebirth details: Participants were asked to indicate the pain relief methods they used, including movement and positional changes, with the option to specify additional techniques. To explore the impact of mobility on birth choices, respondents rated the extent to which their ability to change positions influenced their decision to give birth at home using a five-point Likert scale (Not at all to Extremely). They also reported whether they were able to move freely during labor (Yes, No, or Partially). Additionally, the section examined experiences related to decision-making and respect, where participants rated their agreement with statements regarding healthcare provider inclusion in decision-making, the adequacy of information provided, and the extent to which their choices were respected in both home and hospital birth settings. Lastly, participants were asked to report the duration of their homebirth and the number of vaginal exams they underwent, allowing for further analysis of procedural differences in homebirth experiences; c) MADM: The MADM scale assesses the degree to which women felt autonomous in making decisions about their care during childbirth. It provides critical insights into empowerment and informed participation of mothers in the childbirth process [13]; d) MORi: The MOR index evaluates perceived respect from healthcare providers during childbirth. This includes dimensions related to decision-making, provider behavior, and the ability to express concerns or ask questions during care [14]; e) BSS-R: The BSS-R captures emotional and physical aspects of childbirth satisfaction. It includes dimensions like the quality of care provided, management of stress during labor, and the mother’s personal attributes, providing a comprehensive measure of maternal satisfaction [15]; and f) MIST: We first obtained the necessary permission to convert the MIST scale to Greek by contacting the developers of the scale via email. The scale was translated into the Greek language by an expert who was fluent in both languages. The translated scale was evaluated by experts, and corrections were made in line with their opinions. The translated scale was then translated back into English by individuals who were proficient in the language. Items based on the MIST scale are included to identify specific incidents of mistreatment, such as privacy violations, verbal abuse, or neglect, during labor and delivery. This section helps to identify areas where maternal care quality may have fallen short.

The part of Demographics included 15 questions, of which 7 were open-ended (e.g., age, city of residence) and 8 were closed-ended (e.g., sex, marital status). The section titled First Homebirth Information consisted of 10 questions, primarily open-ended, addressing variables such as gestational week, income, and professional status during the first homebirth. The Homebirth Questionnaire section comprised 5 questions, 2 open-ended (e.g., pain relief methods) and 3 closed-ended (e.g., ability to change positions). The MADM scale contained 7 closed-ended Likert-type items. The MORi included 14 closed-ended Likert-scale questions divided into 3 subsections (A, B, and C). Lastly, the BSS-R consisted of 10 closed-ended Likert-scale items, each accompanied by an optional open-ended comments field.

All measurement tools used in this study, including the MADM scale, the MORi, the BSS-R, and the MIST, were used with written permission obtained via email from the original authors. These tools were employed in accordance with the terms of use specified by their developers and are cited appropriately in this manuscript.

Ethical considerations

The study protocol was approved by the Research Ethics Board of the University of West Attica (protocol number 77034/01-09-2023).

Statistical analysis

Using the Kolmogorov-Smirnov test, the distributions of quantitative variables were assessed for normality. For variables that followed a normal distribution, mean values and standard deviations (SD) were used for their description. For those that did not follow a normal distribution, medians and interquartile ranges (IQR) were additionally used. Absolute (N) and relative (%) frequencies were employed to describe qualitative variables. The non-parametric Mann-Whitney test was used to compare quantitative variables between two groups, while Spearman's correlation coefficient was applied to assess the relationship between two quantitative variables. The Wilcoxon signed-rank test was utilized to compare scales related to respect in maternity wards versus home settings. The reliability of the questionnaires and their subscales was assessed using Cronbach's alpha reliability coefficient.

To identify independent factors associated with satisfaction scales during childbirth (BSS-R), maternal autonomy (MADM), and respect during home childbirth (MORi), a linear regression analysis with stepwise inclusion/exclusion was performed. This analysis yielded dependency coefficients (β) and their standard errors (SE). When the dependent variable's distribution was not normal, its logarithm was used in the analysis. Logistic regression analysis with stepwise inclusion/exclusion was conducted to identify factors related to the experience of mistreatment by healthcare professionals, yielding odds ratios (OR) with their 95% confidence intervals (95% CI). Significance levels were two-tailed, with statistical significance set at 0.05. The statistical analysis was performed using IBM SPSS Statistics for Windows, version 26.0 (IBM Corp., Armonk, NY).

## Results

Demographics

The sample consists of 162 women with a mean age of 36.4 years (standard deviation =5.4 years). One hundred and fifty-three (94.4%) of the participants had Greek nationality, and 87 (54%) lived in Attica. Also, 95 (50.6%) participants had a bachelor’s degree, 133 (82.1%) were married, and most of them, 76 (46.9%), had two children.

Table 1 summarizes the demographic characteristics of the study population.

**Table 1:** Demographic characteristics of the study population

Parameter		Ν	%
Age, Mean value (standard deviation) (years)		36.4 (5.4)	
Nationality	Other	9	5.6
	Greek	153	94.4
Prefecture of residence: Attica	No	74	46
	Yes	87	54
Educational status	No education	1	0,6
	High school	7	4.3
	Technical school	3	1.9
	Vocational school for two years of training	13	8
	Bachelor’s degree	95	50.6
	Master’s degree	51	31.5
	Doctorate	5	3.1
Marital status	Married	133	82.1
	Single	5	3.1
	Divorced	4	2.5
	Cohabitation agreement	15	9.3
	Cohabitation	5	3.1
Number of children	1	28	17.3
	2	76	46.9
	3	42	25.9
	4	8	4.9
	5	5	3.1
	6	3	1.9

Comparison of respect toward mothers during childbirth at home and in maternity care settings

One hundred and two women reported having prior childbirth experience both in a maternity hospital and at home, and have completed the respective questionnaires for both settings.

Table 2 illustrates the descriptive statistics for the dimensions and the overall MORi scale, both for home and maternity hospitals.

**Table 2:** Descriptive statistics for the dimensions and the overall MORi scale, both for home and maternity hospitals IQR: interquartile range; SD: standard deviation; MORi: Mothers on Respect Index

Dimension	Minimal value	Maximal value	Mean value (SD)	Median value (IQR)	Cronbach's α
Dimension about respect in making decisions about my pregnancy or childbirth care (home)	19.0	42.0	37.2 (5.3)	39 (35 ─ 42)	0.85
Dimension regarding the behavior of a doctor or midwife during pregnancy (home)	4.0	24.0	22.5 (4)	24 (24 ─ 24)	
Dimension regarding the possibility of asking questions and discussing concerns with the doctor or midwife during pregnancy (home)	3.0	18.0	16.2 (3.3)	18 (15 ─ 18)	
MORi (home)	43.0	84.0	74.4 (8.8)	78 (70 ─ 81)	
Dimension about respect in making decisions about my pregnancy or childbirth care (maternity hospital)	7.0	42.0	23.3 (9.6)	22 (16 ─ 29)	0.90
Dimension regarding the behavior of a doctor or midwife during pregnancy (maternity hospital)	9.0	24.0	20.7 (3.7)	22 (18 ─ 24)	
Dimension regarding the possibility of asking questions and discussing concerns with the doctor or midwife during pregnancy (maternity hospital)	3.0	18.0	10.8 (4.4)	10.5 (8 ─ 14)	
MORi (maternity hospital)	28.0	84.0	56.4 (13.7)	55 (46 ─ 64)	

Subsequently, a comparison was made between the MORi scores in home and maternity hospital settings, revealing that scores were higher across all dimensions and the overall scale for respect provided at home (p<0.001 for all comparisons) (Table 3).

**Table 3:** Levels of respect in the maternity hospital and at home

Level of respect	Ν (%)		p
	Maternity hospital	Home	-
Very low level of respect	1 (1.0)	0 (0.0)	<0.001
Low level of respect	31 (30.4)	3 (2.9)	<0.001
Moderate level of respect	49 (48.0)	14 (13.7)	<0.001
High level of respect	21 (20.6)	85 (83.3)	<0.001

In the maternity hospital, 49 (48%) of women experienced moderate respect, whereas the corresponding number at home was 14 (13.7%) (p<0.001). Regarding respect at home, 85 (83.3%) reported receiving high respect, compared to 21 (20.6%) for the maternity hospital (p<0.001).

Spearman correlations between the dimensions of the MORi in the maternity hospital and those for home childbirth

Table 4 presents Spearman correlations between the dimensions of the MORi in the maternity hospital and those for home childbirth.

**Table 4:** Spearman correlations between the dimensions of the MORi in the maternity hospital and those for home childbirth MORi: Mothers on Respect Index

Parameter		Dimension about respect in making decisions about my pregnancy or childbirth care (maternal hospital)	Dimension regarding the behavior of a doctor or midwife during pregnancy (maternity hospital)	Dimension regarding the possibility of asking questions and discussing concerns with the doctor or midwife during pregnancy (maternity hospital)	MORi (home)
Dimension about respect in making decisions about my pregnancy or childbirth care (home)	rho	-0.08	0.20	-0.02	0.04
-	P	0.417	0.044	0.831	0.683
Dimension regarding the behavior of a doctor or midwife during pregnancy (home)	rho	0.01	0.30	0.01	0.07
-	P	0.990	0.002	0.973	0.479
Dimension regarding the possibility of asking questions and discussing concerns with the doctor or midwife during pregnancy (home)	rho	-0.03	0.29	0.13	0.12
-	P	0.762	0.003	0.206	0.228
MORi (home)	rho	0.23	0.43	0.19	0.31
-	P	0.018	<0.001	0.061	0.001

Both the dimensions and the overall scales for evaluating respect in the maternity hospital and at home were found to be significantly and positively correlated in most cases. Therefore, women who perceived higher respect in the maternity hospital also tended to have a more positive perception of the respect they received at home.

BSS-R, MADM, and their correlation

Table 5 presents the descriptive statistics for the dimensions and the overall scale of BSS-R.

**Table 5:** Descriptive statistics for the dimensions and the overall scale of BSS-R IQR: interquartile range; SD: standard deviation; BSS-R: Birth Satisfaction Scale-Revised

Dimension	Minimal value	Maximal value	Mean value (SD)	Median value (IQR)	Cronbach's α
Quality of care provided	0.00	16.00	13.5 (3.1)	14 (13 ─ 16)	0.82
Personal attributes of women	0.00	8.00	5.9 (1.9)	6 (5 ─ 7)	0.72
Stress management during birth	2.00	16.00	12.3 (2.9)	13 (11 ─ 14)	0.71
Overall BSS-R scale	3.00	40.00	31.8 (6.5)	33 (28 ─ 36)	0.83

The "Quality of Care Provided" dimension ranged from 0 to 16 points, with a mean score of 13.5 points (SD = 3.1 points). The "Personal Attributes" dimension ranged from 0 to 8 points, with a mean score of 5.9 points (SD = 1.9 points), while the "Stress Management During Birth" dimension ranged from 2 to 16 points, with a mean score of 12.3 points (SD = 2.9 points). The overall satisfaction scale ranged from 3 to 40 points, with a mean score of 31.8 points (SD = 6.5 points). The MADM scale score ranged from 16 to 42 points, with a mean score of 37 points (SD = 5.5 points).

Table 6 presents the Spearman correlation between BSS-R and the MADM scales’ scores.

**Table 6:** Spearman correlation for BSS-R and the MADM scores BSSR: Birth Satisfaction Scale-Revised; MADM: Mother's Autonomy in Decision Making

Dimension of BSS-R		MADM scale score
Quality of care provided	rho	0.39
	P	<0.001
Personal attributes of women	rho	0.30
	P	<0.001
Stress management during birth	rho	0.24
	P	0.002
Overall birth satisfaction scale	rho	0.35
	P	<0.001

The MADM scale score was positively and significantly correlated with all dimensions and the overall BSS-R scale score (p<0.05). This indicates that greater autonomy was associated with higher satisfaction with childbirth.

Responses to MIST

Table 7 presents the responses to the MIST scale.

**Table 7:** Responses to MIST MIST: Mistreatment in Childbirth

MIST questions		Ν	%
Your private or personal information was shared without your consent		1	0.8
Your physical privacy was violated (i.e., being uncovered or having people in the delivery room without your consent)		2	1.6
Health care providers (doctors, midwives, or nurses) shouted at or scolded you		9	7.4
Health care providers threatened to withhold treatment or to force you to accept treatment you did not want		2	1.6
Health care providers threatened you in any other way		3	2.5
Health care providers ignored you, refused your requests for help, or failed to respond to requests for help in a reasonable amount of time.		5	4.1
You experienced physical abuse (including aggressive physical contact, inappropriate sexual conduct, a refusal to provide anesthesia for an episiotomy, etc.)		1	0.8
Have you experienced at least one of the above behaviors?	No	107	88.4
	Yes	14	11.6

Overall, the vast majority of women (n=107, 88.4%) reported not experiencing any form of mistreatment during childbirth, indicating that most received care that respected their dignity, autonomy, and rights. Only 14 (11.6%) reported at least one instance of mistreatment. Specifically, 1 (n=0.8%) participant indicated that her private information had been shared without consent or that they had experienced physical abuse, such as aggressive contact, inappropriate sexual behavior, or denial of anesthesia for an episiotomy. Similarly, 2 (1.6%) reported a violation of physical privacy or coercion to accept unwanted treatment. Although 9 (7.4%) of women reported being shouted at or scolded by healthcare providers, and smaller proportions indicated feeling ignored (5, 4.1%) or threatened (3, 2.5%), the findings overall highlight that more than 8 in 10 women experienced respectful and supportive care during childbirth.

Correlation of BSS-R and MADM with MORi

Table 8 presents the correlation of BSS-R and MADM scores in the home and in the maternity hospital with the MORi score.

**Table 8:** Correlation of BSS-R and MADM scores in home and in maternity hospital with MORi score BSSR: Birth Satisfaction Scale-Revised; MADM: Mother's Autonomy in Decision Making; MORi: Mothers on Respect Index

Dimension of MORi		Overall BSS-R score	MADM scale score
Dimension about respect in making decisions about my pregnancy or childbirth care (home)	rho	-0.07	-0.04
	P	0.511	0.679
Dimension regarding the behavior of a doctor or midwife during pregnancy (home)	rho	0.07	0.15
	P	0.396	0.065
Dimension regarding the possibility of asking questions and discussing concerns with the doctor or midwife during pregnancy (home)	rho	0.01	0.01
	P	0.939	0.940
MORi (home)	rho	0.02	0.03
	P	0.880	0.731
Dimension about respect in making decisions about my pregnancy or childbirth care (maternity hospital)	rho	-0.09	-0.03
	P	0.361	0.797
Dimension regarding the behavior of a doctor or midwife during pregnancy (maternity hospital)	rho	0.06	0.04
	P	0.529	0.724
Dimension regarding the possibility of asking questions and discussing concerns with the doctor or midwife during pregnancy (maternity hospital)	rho	-0.11	-0.21
	P	0.291	0.032
MORi (maternity hospital)	rho	-0.06	-0.06
	P	0.534	0.523

The BSS-R score and the MADM score were not found to be significantly correlated with the MORi scale score.

Association of BSS-R, MADM, and MORi scores with MIST score

Subsequently, an analysis was conducted to determine whether the BSS-R, MADM, and MORi scales differed based on whether women had experienced mistreatment.

Table 9 presents the association of BSS-R, MADM, and MORi scores with the MIST score.

**Table 9:** Association of BSS-R, MADM, and MORi scores with MIST score BSSR: Birth Satisfaction Scale-Revised; MADM: Mother's Autonomy in Decision Making; MORi: Mothers on Respect Index; IQR: interquartile range; SD: standard deviation

Dimension	Have you experienced mistreatment from a healthcare provider? (MIST)				P
	No		Yes		
	Mean value (SD)	Median (IQR)	Mean value (SD)	Median (IQR)	
Dimension about respect in making decisions about my pregnancy or childbirth care (home) (MOR)	37.5 (4.7)	39 (35 ─ 42)	34.6 (8.9)	38 (26 ─ 42)	0.585
Dimension regarding the behavior of a doctor or midwife during pregnancy (home) (MOR)	22.5 (3.9)	24 (24 ─ 24)	19.3 (7.4)	24 (18 ─ 24)	0.045
Dimension regarding the possibility of asking questions and discussing concerns with the doctor or midwife during pregnancy (home) (MOR)	16.1 (3.3)	18 (15 ─ 18)	16.1 (3.5)	18 (17 ─ 18)	0.821
MORi (home)	74.3 (8.1)	76.5 (70 ─ 80)	69 (12.3)	75 (59 ─ 80)	0.266
Dimension about respect in making decisions about my pregnancy or childbirth care (maternity hospital) (MOR)	21.5 (9.5)	21.5 (12 ─ 29)	21.5 (6.4)	22 (18 ─ 25)	0.945
Dimension regarding the behavior of a doctor or midwife during pregnancy (maternity hospital) (MOR)	20 (3.7)	20 (18 ─ 24)	20.5 (4.4)	23 (16 ─ 24)	0.574
Dimension regarding the possibility of asking questions and discussing concerns with the doctor or midwife during pregnancy (maternity hospital) (MOR)	10.1 (4.3)	10 (7 ─ 13)	12.4 (4.1)	12 (10 ─ 15)	0.096
MORi (maternity hospital)	53.7 (13.2)	51 (43 ─ 63)	55.2 (12.4)	55 (52 ─ 63)	0.517
Quality of care provided (BSS-R)	13.5 (3.4)	15 (13 ─ 16)	12.1 (3.1)	12.5 (11 ─ 14)	0.027
Personal attributes of women (BSS-R)	5.9 (1.8)	6 (5 ─ 7)	5.1 (2.2)	6 (3 ─ 7)	0.197
Stress management during birth (BSS-R)	12.3 (3)	13 (11 ─ 15)	11.5 (3)	11.5 (9 ─ 15)	0.287
Overall BSS-R scale score	31.7 (6.9)	33 (29 ─ 36)	28.8 (5.9)	27.5 (24 ─ 34)	0.045
Overall MADM scale score	37.2 (5.2)	38 (35 ─ 42)	31.7 (7.7)	34.5 (28 ─ 37)	0.005

Women who reported experiencing mistreatment by a healthcare provider were less satisfied with the behavior of doctors or midwives during pregnancy (at home) compared to those who had not experienced such mistreatment (p=0.045). Additionally, the dimension related to the quality of care provided and the overall BSS-R scale were found to differ depending on whether women had experienced abusive behavior (p=0.027). Specifically, those who had experienced mistreatment reported lower quality of care and overall birth satisfaction (p=0.045). Finally, women who had experienced mistreatment also reported less autonomy in decision-making (p=0.005).

Multivariate analysis for factors influencing birth satisfaction, maternal autonomy, and respect for women in home birth settings

To identify the factors independently associated with birth satisfaction, maternal autonomy, and respect for women in home birth settings, multivariate linear regression analyses were conducted. The dependent variables were the scores on these scales and their dimensions, while the independent variables included women's demographic characteristics and childbirth-related factors.

Dimension of Quality of Care Provided (BSS-R)

The quality of care provided was significantly influenced by the information given to women by the midwifery team. Better explanations about home childbirth procedures were associated with higher evaluations of the quality of care provided (β= 0.815, SE= 0.283, P=0.005).

Dimension of Personal Attributes of Women (BSS-R)

The ability to comfortably and freely choose a position for childbirth was independently associated with women’s coping capacity. Greater freedom in choosing a birth position was related to greater coping ability (β= 0.035, SE= 0.016, b= 0.171, P= 0.034).

Dimension Related to Stress Management During Childbirth (BSS-R)

The number of vaginal examinations and the duration of labor were independently associated with stress management during childbirth. A greater number of vaginal examinations was linked to lower stress management scores (β= -0.020, SE= 0.008, P= 0.010). Additionally, longer labor durations were associated with decreased stress management (β= -0.002, SE= 0.001, P= 0.033).

Overall Birth Satisfaction Score (BSS-R)

Two factors were significantly associated with the overall BSS-R score: a) The ability to comfortably and freely choose a birth position was linked to greater satisfaction (β= 0.022, SE= 0.008, b= -0.202, P= 0.010); b) The duration of home birth was negatively associated with birth satisfaction. Longer labor resulted in lower satisfaction (β= -0.087, SE= 0.041, b= -0.164, P= 0.034).

Maternal Autonomy in Decision Making (MADM) Scale

The adequacy of explanations provided by the midwifery team about home birth procedures was significantly associated with the maternal autonomy score. Women who received better explanations reported higher autonomy during childbirth (β= 0.046, SE= 0.006, b= 0.521, P< 0.001).

Respect for Women in Home Birth Settings (MORi Score)

Greater comfort was associated with higher respect from healthcare professionals during pregnancy or childbirth at home (β= 0.068, SE= 0.013, P< 0.001). Additionally, comfort in asking questions and discussing concerns with doctors or midwives was linked to greater respect (β= 0.036, SE= 0.011, P= 0.002).

Experience of Mistreatment by Healthcare Providers (MIST Score)

Women who were better informed about the potential risks of home birth were less likely to report experiencing mistreatment by a healthcare provider (OR= 0.47, 95% CI= 0.27-0.80, P= 0.006).

## Discussion

The findings of this study provide valuable insights into the experiences of respect, autonomy, and satisfaction among women during childbirth, comparing home and hospital settings. Using validated tools such as the MORi, the MADM scale, and the BSS-R, this research highlights critical differences in care quality across these environments. The study’s results have significant implications for improving maternity care practices and policies, ensuring that maternal dignity and autonomy remain central to childbirth experiences.

Women reported significantly higher scores on respect (MORi) and satisfaction (BSS-R) in home birth settings compared to hospitals. In particular, the study revealed that 83.3% of women experienced high respect in home births, whereas only 20.6% reported similar respect levels in hospital settings. These findings underscore the personalized and autonomy-focused nature of home births, where women often have greater control over their birthing experience.

The positive correlations between autonomy (MADM) and satisfaction (BSS-R) further emphasize the role of informed decision-making in shaping childbirth experiences. Women who felt empowered to make decisions about their care were more likely to express satisfaction, particularly in home birth settings where autonomy was more frequently facilitated.

The study found that respectful behavior and clear communication by healthcare professionals were pivotal in influencing maternal satisfaction and autonomy. Women who perceived midwives as supportive and respectful during childbirth reported higher satisfaction scores. Specifically, being adequately informed about home birth procedures was significantly associated with both higher satisfaction (BSS-R) and autonomy (MADM).

While the overall prevalence of mistreatment was low (11.6%), the experiences reported, such as being shouted at or scolded (7.4%) or privacy violations (1.6%), had notable negative impacts on satisfaction and autonomy scores. These findings highlight the critical need to address disrespectful maternity care practices, particularly in hospital settings, where structured protocols may inadvertently de-prioritize individualized care.

Interestingly, the study found that women who perceived higher respect in hospital settings tended to report more positive experiences in home births as well. This correlation suggests that prior positive experiences with respectful care can shape expectations and perceptions of subsequent childbirth experiences.

The findings align with prior studies emphasizing the importance of respectful maternity care in fostering positive emotional and psychological outcomes postpartum. Research has consistently shown that autonomy and respect are integral to maternal satisfaction, and these elements are more readily achieved in less structured, individualized care environments such as home births [3,16-19].

However, the study also highlights gaps in hospital-based maternity care. While hospitals are equipped to handle complications, their structured protocols may limit women’s autonomy and personalized care, as evidenced by lower MORi and MADM scores. These findings resonate with global calls to integrate respectful maternity care principles into institutional settings [20].

Implications for policy and practice

The study’s findings advocate for adopting elements of home birth practices in hospital settings. Ensuring women’s autonomy, providing clear information, and fostering supportive environments can enhance satisfaction and reduce negative perceptions of hospital births. Targeted training programs for midwives, obstetricians, and nurses can help address gaps in respectful care practices. Emphasizing communication skills, shared decision-making, and cultural sensitivity is essential to improving maternal experiences across all settings. Incorporating tools such as MORi, MADM, and BSS-R into routine practice can help healthcare systems monitor and improve maternity care quality. Regular assessments can identify areas where interventions are needed, ensuring consistent delivery of respectful and individualized care.

Strengths

The study employed validated and culturally adapted tools (MORi, MADM, BSS-R) to comprehensively assess maternal experiences, ensuring robust and reliable data. By focusing on women with experiences in both home and hospital births, the study provides unique insights into the comparative quality of care. With a significant sample size of 162 women, the study’s findings are generalizable to similar populations.

Limitations

The reliance on self-reported data introduces the potential for recall bias, particularly regarding childbirth experiences that occurred years earlier, some dating as far back as 2010. Recruitment through social media groups may have led to selection bias, potentially attracting women with particularly strong views (either positive or negative) about home births, thus limiting the generalizability of findings to the broader maternal population. The exclusive inclusion of women with at least one home birth experience further narrows the scope, excluding perspectives of those who gave birth exclusively in hospital settings. Moreover, the study’s findings are specific to the Greek healthcare context and may not be directly applicable to other cultural or healthcare systems with different maternity care structures or birth practices. While the sample included some demographic diversity, it remained predominantly Greek, restricting the ability to explore how factors such as ethnicity, socioeconomic status, or immigration status shape maternity experiences. The online, self-administered survey format may have excluded women without reliable internet access or those less digitally literate, further reducing representativeness. Additionally, the study would benefit from greater methodological transparency-particularly regarding survey response rates, sampling strategy, and exclusion criteria. Finally, although multivariate regression analyses were conducted, the potential influence of unmeasured or uncontrolled confounding factors such as prior traumatic birth experiences, parity, and mental health status may have affected the observed associations.

Recommendations for future research

Future studies should employ longitudinal designs to assess the long-term effects of respectful and autonomous maternity care on both maternal and child health outcomes. Examining the impact of care experiences on postpartum mental health, breastfeeding success, and early childhood development will provide valuable insights into the sustained benefits of autonomy in childbirth. Additional research is needed to explore how socioeconomic status, cultural beliefs, and systemic healthcare structures shape women's maternity care experiences. Investigating disparities in access to autonomous and respectful care can inform policies that promote equitable maternal health services across diverse populations. There is a need for rigorous evaluation of interventions aimed at reducing mistreatment and improving autonomy in hospital-based maternity care. Future studies should assess the effectiveness of training programs, patient-centered care models, and policy reforms in enhancing maternal satisfaction and reducing negative childbirth experiences in institutional settings. Investigating the perspectives of obstetricians, midwives, and nurses can help identify key barriers and facilitators to delivering respectful and autonomous care. Understanding providers' challenges, perceptions, and institutional constraints will be essential for designing targeted interventions that foster a more patient-centered approach to maternity care.

## Conclusions

This study highlights significant disparities in respect, autonomy, and satisfaction between home and hospital births. The findings underscore the critical importance of personalized, respectful, and autonomous care in shaping positive maternal experiences. By integrating elements of home birth practices into hospital settings and prioritizing respectful maternity care across all environments, healthcare systems can ensure that every woman’s childbirth experience is dignified and empowering.
